# Desaminotyrosine promotes tuft cell expansion and integrates intestinal type 2 immunity

**DOI:** 10.1128/mbio.03289-25

**Published:** 2026-01-23

**Authors:** Wanqing Zang, Zhou Zhou, Yantong Shen, Bei Zhang, Xinyu Chen, Wenjing Yue, Xiao Li, Yaotian Cai, Junyu Chen, Jiawei Bian, Leyuan Huang, Hongcui Li, Yang Dai, Huan Yang

**Affiliations:** 1 Xuzhou Key Laboratory of Laboratory Diagnostics, School of Medical Technology, Xuzhou Medical University 38044 , Xuzhou, Jiangsu, China; 2 Jiangsu Institute of Parasitic Diseases 608817, Wuxi, Jiangsu, China; 3 Wuhan Children's Hospital (Wuhan Maternal and Child Healthcare Hospital), Tongji Medical College, Huazhong University of Science & Technology, Wuhan, Hubei, China; 4 Jiangsu Provincial Medical Key Laboratory, Jiangsu Provincial Key Laboratory on Parasitic and Vector Control Technology, National Health Commission Key Laboratory of Parasitic Disease Control and Prevention, Jiangsu Institute of Parasitic Diseases 608817, Wuxi, Jiangsu, China; Cornell University College of Veterinary Medicine, Ithaca, New York, USA

**Keywords:** gut microbiota metabolites, Intestinal epithelial cells (IECs), type 2 immunity, microbiome

## Abstract

Intestinal microbiota are essential for maintaining the host’s immune homeostasis, but the mechanism is not fully understood. While microbial metabolite desaminotyrosine (DAT) is recognized for its protective role in viral immunity, its potential involvement in anti-parasitic defense remains unexplored. Here, we demonstrate that DAT orchestrates tuft cell hyperplasia and subsequent type 2 immunity, establishing critical defense against helminth infection. Mechanistically, DAT-mediated intestinal epithelial remodeling requires histone deacetylase 3 (HDAC3), as pharmacological inhibition of this epigenetic regulator abrogates both tuft cell expansion and impairs type 2 immune responses. Collectively, our findings not only explore DAT novel effects in anti-parasitic defense but also reveal a pathway whereby the small molecule metabolites calibrate intestinal type 2 immunity.

**IMPORTANCE:**

A small molecule metabolite DAT drives tuft cell hyperplasia and type 2 immunity in the small intestine. DAT-mediated tuft cell hyperplasia depends on HDAC3 and an intact microbiota; our findings reveal how small molecule metabolites fine-tune intestinal type 2 defenses against parasites.

## INTRODUCTION

Intestinal epithelium has illuminated the diverse functions and importance of intestinal epithelial cells, which serve as essential barriers and immune regulators (1). These cells are constantly influenced by the gut microbiota and its metabolites, which play a crucial role in modulating immune responses and maintaining intestinal homeostasis (2–5).

Among the various epithelial cell types, tuft cells represent a specialized population with a distinctive “brush-like” morphology. These cells are known for their sensory functions, responding to a range of external stimuli, including gut microbiota and its metabolites (6, 7). Tuft cells play a key role in immune regulation, particularly in the small intestine, where they secrete IL-25 (also known as IL-17E) (8, 9). Tuft cell-derived IL-25 is essential for activating type 2 innate lymphoid cells (ILC2s) (10), which, similar to type 2 helper T cells (Th2), produce type 2 immune factors like IL-4 and IL-13. These factors are vital for the body’s anti-helminth response, as they help coordinate the immune system’s defense against parasitic infections. IL-13, in turn, regulates tuft cell proliferation, creating a feedback loop that supports epithelial regeneration and ensures the rapid renewal of the intestinal lining (11). This regenerative process, which takes less than a week, enables the intestine to swiftly respond to parasitic challenges and restore homeostasis.

Tuft cells play pivotal roles in gut homeostasis and immune regulation (12). Their ability to sense microbiota-derived signals and orchestrate immune responses highlights the complex interactions between the gut epithelium, the immune system, and the microbiome. It has been established that the metabolite succinate promotes tuft cell proliferation and induces intestinal type 2 immunity, whereas butyrate exerts opposing effects (13). While efforts are underway to identify the factors that drive tuft cell hyperplasia, the mechanisms governing tuft cell development and function are still not well understood. Understanding these interactions is essential for developing novel therapeutic strategies for diseases involving immune dysregulation or parasitic infections.

DAT, a flavonoid and degradation product of amino acids metabolized by common gut bacteria, has recently garnered attention for its potential role in modulating immune responses (14, 15). DAT has been implicated in combating influenza virus infections and alleviating intestinal inflammation, primarily through type I interferon (16). However, despite these promising findings, research on DAT is still in its infancy, and its deeper mechanisms and clinical relevance remain largely unexplored.

Here, we aim to investigate the potential of DAT as a tuft-sensing stimulator to enhance immune responses. Specifically, we explore its ability to promote tuft cell proliferation and stimulate IL-25-ILC2 circulation, both of which are crucial for managing parasitic infections (17). By modulating immune pathways, DAT may not only help treat parasitic infections but also alleviate related symptoms and complications. Furthermore, this research seeks to uncover the specific immunomodulatory effects of DAT, shedding light on its broader therapeutic potential in parasitic diseases. Given DAT’s emerging role in immune regulation, further investigation into the mechanistic aspects of DAT could lead to the development of innovative therapeutic strategies targeting immune dysregulation. This holds significant clinical promise for treating intestinal infections and other immune-related conditions.

## RESULTS

### DAT promotes intestinal tuft cell expansion

**Fig 1:**
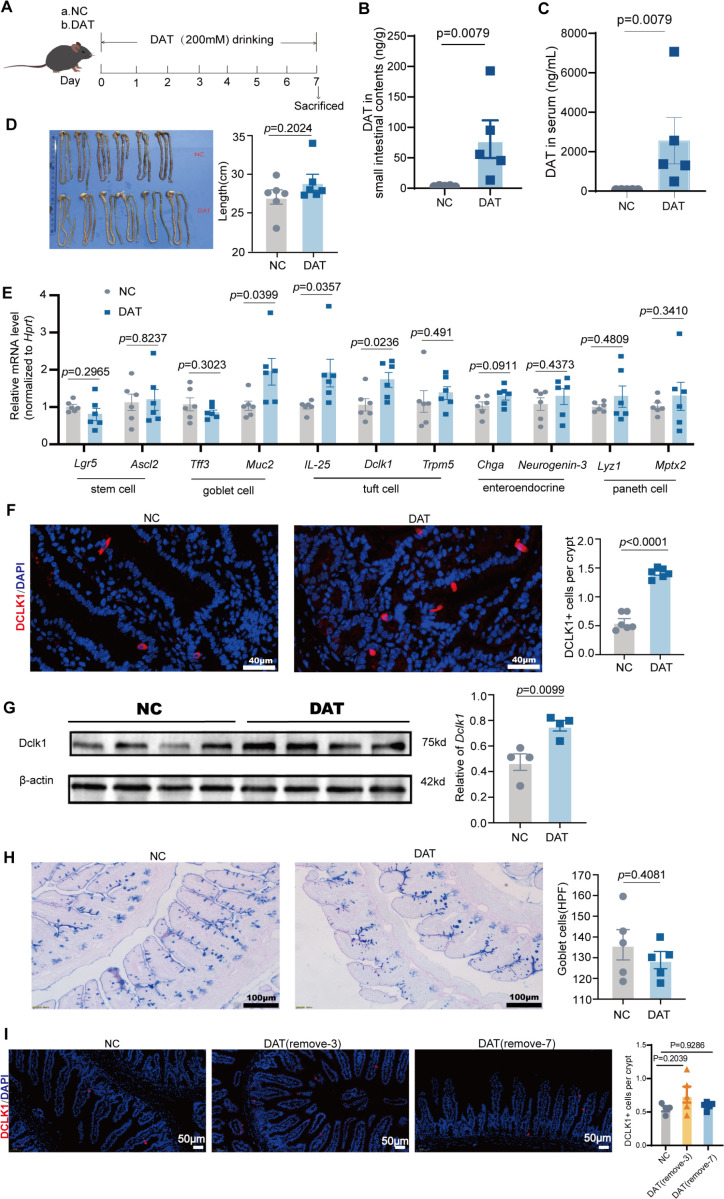
DAT promotes intestinal tuft cell expansion. (**A**) Schematic of the experimental design. (**B and C**) Targeted metabolomics analysis of DAT levels in mouse small intestinal contents, serum. (**D**) Gross morphology and length measurements of the small intestine in mice (*n* = 6 per group). (**E**) The *Lgr5, Ascl2, Tff3, Muc2, IL-25, Dclk1, Trpm5, Chga*, *Neurogenin-3, Lyz1,* and *Mptx2* levels in the ileocecal region of the small intestine were determined using reverse transcription-quantitative polymerase chain reaction (RT-qPCR) (*n* = 6 per group). (**F**) Fluorescence staining of tuft cells (DCLK1+, red) in ileum and DCLK1+ tuft cells per crypt (*n* = 6 per group). Scale bar: 40 μm. (**G**) Western Blotting analysis of small intestinal tissue: Dclk1 and β-actin staining (*n* = 4 per group). (**H**) Combined Alcian Blue-periodic acid Schiff (AB-PAS) staining was performed to detect goblet cells, average number of goblet cells per high-power field (HPF) (*n* = 6 per group). Scale bar: 100 μm. (**I**) Fluorescence staining of tuft cells (DCLK1+, red) in ileum and DCLK1+ tuft cells per crypt (*n* = 5 per group). Scale bar: 50 μm. Experiments were repeated independently two times. Data are the mean ± SEM. Statistical significance was determined by two-sided Student’s *t* test (**B–H**) or one-way ANOVA (**I**).

Bacterial metabolites, such as succinate and N-undecanoylglycine, promote tuft cell expansion, playing a role in maintaining intestinal homeostasis (12). DAT, a metabolite derived from intestinal bacteria, has been shown to alleviate intestinal inflammatory responses (18). Based on these findings, we hypothesized that DAT might also modulate intestinal epithelial cells. To assess its effects, mice received either 200 mM DAT or vehicle in their drinking water for 1 week (Fig. 1A). The results demonstrated that DAT levels in both intestinal contents and serum were significantly elevated several-fold in mice receiving DAT-supplemented drinking water compared to the vehicle control group (Fig. 1B–C). Almost no DAT was detected in the untreated group, which further confirms that the observed immunological changes were solely attributable to exogenous DAT supplementation. These findings provide a rigorous foundation for subsequent experiments. Gross appearance and size of the small intestine showed no significant differences between DAT-treated and normal control (NC) mice (Fig. 1D).

To determine the regulatory effect of DAT on epithelial cells, we analyzed the signature genes of major small intestinal epithelial cells. Compared with the NC group, DAT treatment did not significantly change the expression of signature genes in stem cells, endocrine cells, and Paneth cells (Fig. 1E). However, gene expression analysis revealed increased tuft cell-related and m*uc2* genes in the DAT-treated group compared to the NC group (Fig. 1E). Consistent with the gene expression of tuft cell, supplement with DAT induced tuft cell hyperplasia in the small intestine (Fig. 1F). In addition, DCLK1 protein expression levels were elevated in the DAT group relative to controls (Fig. 1G). Goblet cells, detected by Alcian Blue-periodic acid Schiff (AB-PAS) staining, were not increased in the DAT group (Fig. 1H). Furthermore, the promotive effect of DAT on tuft cell expansion persisted for 3 days after its withdrawal, indicating the stability of its regulatory function (Fig. 1I). Together, these data demonstrate that DAT administration specifically induces the expansion of intestinal tuft cells.

It has been found that the intestinal metabolite succinate can promote the expansion of tuft cells and a type 2 immune response (13). We further investigated whether DAT intervention affects the levels of succinate. Mass spectrometry analysis revealed that succinate levels in serum and lung tissue remained unchanged following 7-day DAT treatment (Fig. S1B and D). Although DAT increased succinate content in small intestinal contents, the change was not statistically significant (Fig. S1C). A head-to-head comparison of DAT and succinate revealed that both significantly increased intestinal tuft cell numbers, with succinate exhibiting a stronger proliferative effect (Fig. S1E and F). Crucially, our findings suggest that DAT promotes tuft cell expansion through a mechanism independent of succinate, identifying it as a novel metabolite capable of inducing tuft cell proliferation.

### DAT activates tuft cell-dependent type 2 immunity

**Fig 2:**
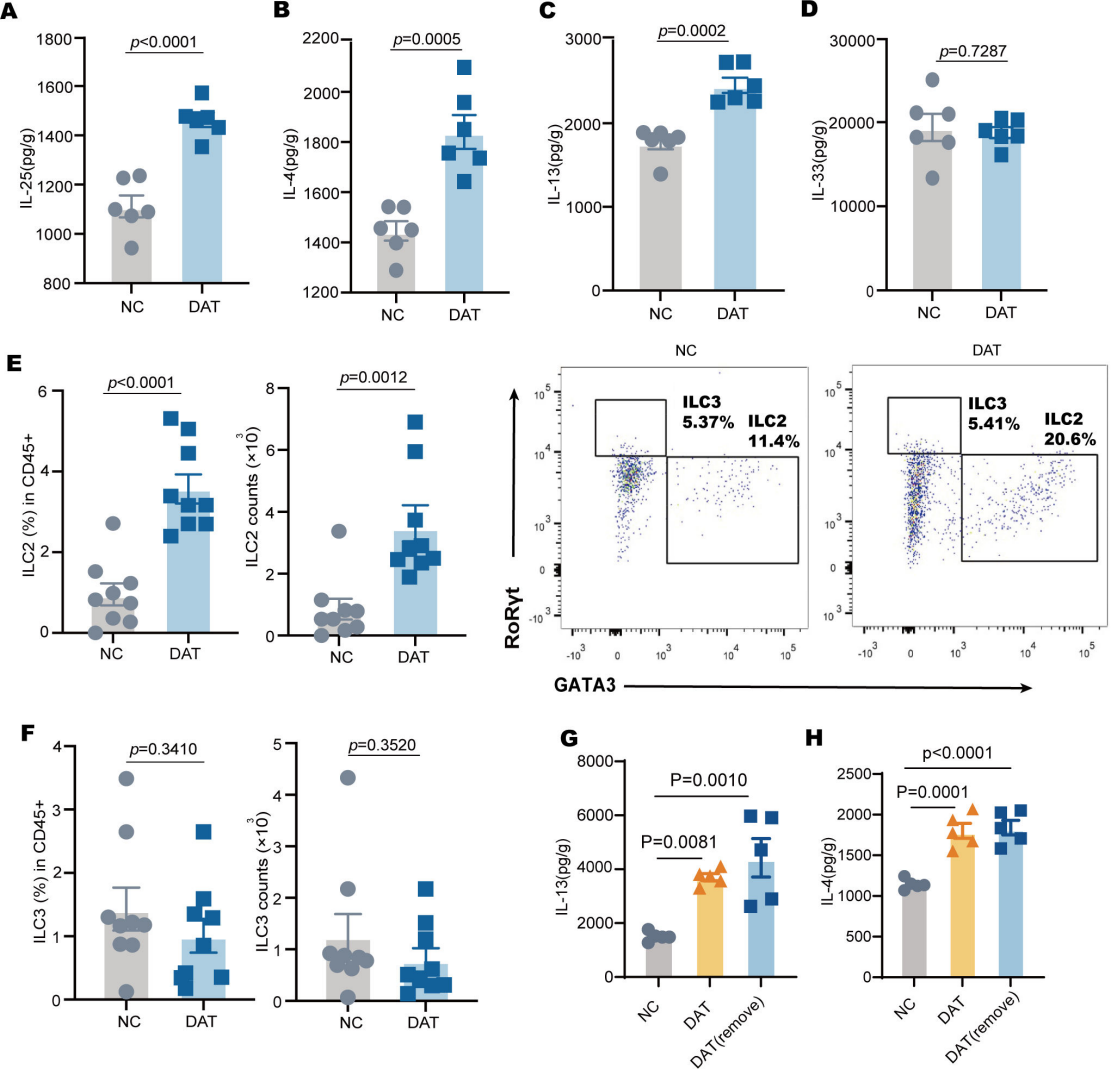
DAT shapes type 2 immunity in the small intestine. (**A–D**) The protein levels of IL-25, IL-4, IL-13, and IL-33 in the small intestine were measured by ELISA (*n* = 6 per group). (**E and F**) ILC2 (CD45+ Lin− CD27+ CD335+ GATA− ROR-ɡ+) and ILC3 (CD45+ Lin− CD27+ CD335+ GATA+ ROR− ɡ−) in the small intestine were measured by flow cytometry. (**G and H**) The protein levels of IL-13, IL-4 in the small intestine were determined by ELISA (*n* = 5 per group). Experiments were repeated independently two times. Data are the mean ± SEM. Statistical significance was determined by two-sided Student’s *t* test (**A–F**) or one-way ANOVA (**G and H**).

Succinic acid can promote tuft cell-dependent type 2 immune responses in the intestine (13). Consequently, we hypothesized that DAT could promote tuft cells expansion and thereby, in addition to IL-25, lead to IL-13 and IL-4 concentrations. To test this, mice were supplemented with DAT in their drinking water. Consistent with our hypothesis, elevated IL-25 protein concentrations were observed in DAT-treated mice compared to untreated controls (Fig. 2A). Levels of IL-4 and IL-13 proteins were also significantly elevated in the ileum of DAT-treated mice (Fig. 2B and C). DAT supplementation also enhanced the accumulation of intestinal group 2 innate lymphoid cells (ILC2s) (Fig. 2E) (19). However, DAT treatment did not change the type 2 immune cytokines IL-33 concentration (Fig. 2D) or ILC3s cell recruitment (Fig. 2F). Together, these data demonstrate that DAT shape type 2 immunity.

Notably, even 3 days after the termination of water intake following the 7-day DAT intervention, intestinal levels of IL-4 and IL-3 in mice remained relatively stable and comparable to those observed during the intervention period (Fig. 2G and H). These results underscore the sustained efficacy and stability of DAT in inducing and maintaining the activation of type 2 immune factors, suggesting a persistent immunomodulatory effect beyond the active treatment phase.

**Fig 3:**
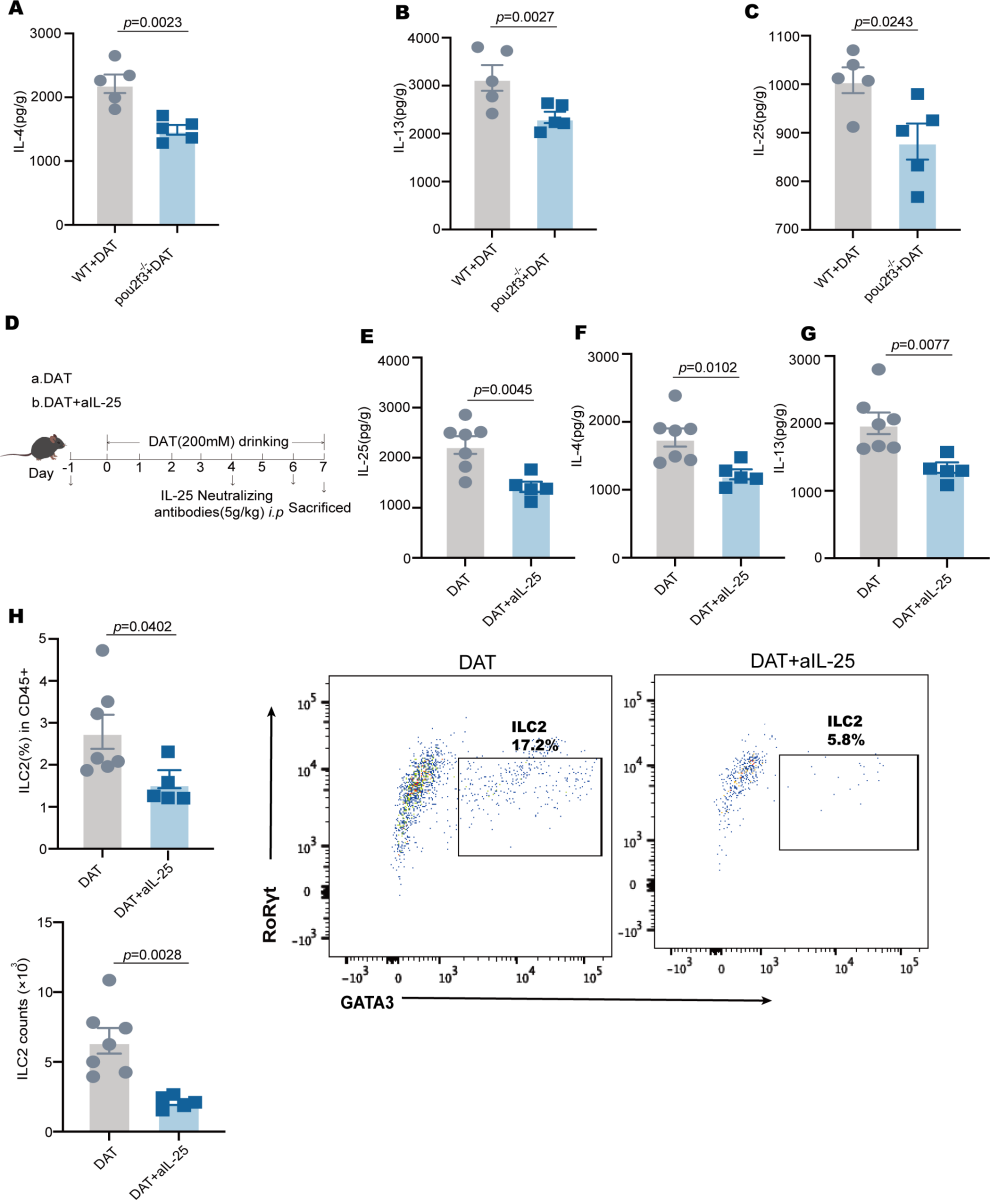
DAT shape tuft cell-dependent type 2 immunity in the small intestine. (**A–C**) IL-4, IL-13, IL-25 in the small intestine were determined by ELISA (*n* = 6 per group). (**D**) A schematic diagram of the experimental approach for IL-25 neutralized with IL-25 neutralizing antibody (aIL-25) in DAT treated model. (**E–G**) The protein levels of IL-25, IL-4, IL-13 in the small intestine were determined by ELISA (*n* = 6 per group). (**H**) The percentage and count of ILC2s (CD45+ Lin− CD27+ CD335+ GATA− ROR-ɡ+) in the small intestine were measured by flow cytometry. Experiments were repeated independently two times. Data are the mean ± SEM. Statistical significance was determined by two-sided Student’s *t* test (**A–C, E–H**).

Previous studies support the role of tuft cells in driving a signaling circuit that initiates the intestinal type 2 immune response (20). *POU2F3* is a lineage-defining transcription factor for tuft cells (21). We then asked whether DAT activation of type 2 immunity depended on tuft cells. In tuft cell-deficient *Pou2f3^−^*^/^*^−^* mice, DAT treatment failed to induce the expression of epithelial IL-4, IL-13, and IL-25 (Fig. 3A through C).

As tuft cell-derived IL-25 is a key initiator of type 2 immunity (9), we hypothesized that DAT activates this pathway through IL-25 secretion by tuft cells (22). To directly assess the role of IL-25 in ILC2 activation during DAT stimulation, we performed IL-25 neutralization experiments (Fig. 3D). Neutralization of IL-25 resulted in a marked reduction in the expression of the type 2 cytokines IL-25, IL-4, and IL-13 within the small intestine, accompanied by decreased ILC2 proliferation (Fig. 3E through H). Neutralization of IL-25 resulted in a marked reduction in the expression of the type 2 cytokines IL-25, IL-4, and IL-13 within the small intestine, accompanied by decreased ILC2 proliferation.

### Gut microbiota dependency of DAT-induced tuft cell proliferation

**Fig 4:**
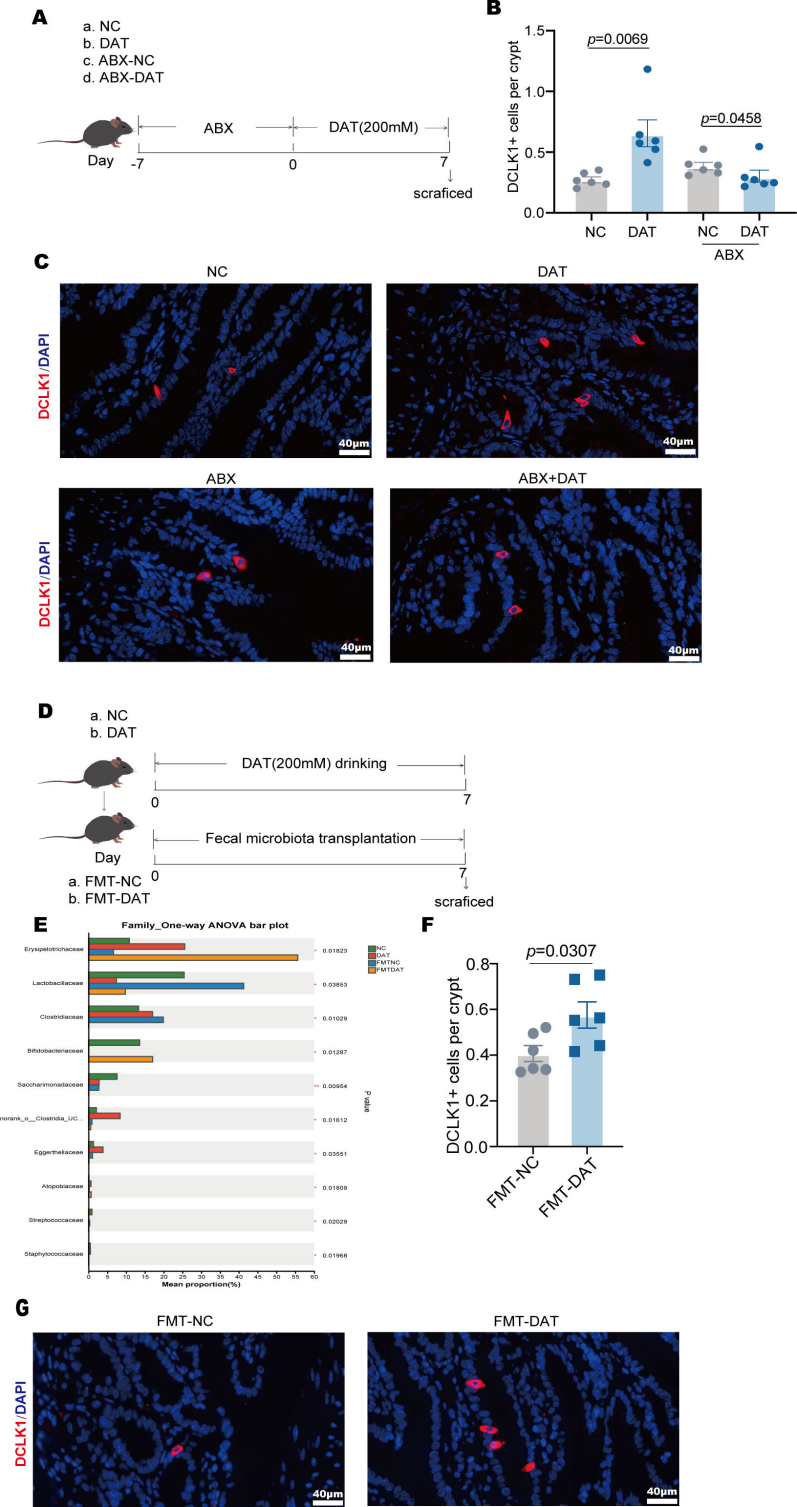
Tuft cell proliferation induced by DAT is dependent on gut microbiota. (**A**) A schematic diagram of the experimental approach of clearing the gut microbiota of mice with mixed antibiotic (ABX) treatment in DAT model. (**B and C**) Immunofluorescence staining of small intestinal tuft cells. Tuft cells were labeled with Dclk1 (red) and counterstained with DAPI (blue) for nuclear visualization. The average number of cluster cells per individual villus was quantified (*n* = 6 per group). Scale bar: 40 μm. (**D**) A schematic diagram of the experimental approach of fecal microbiota transplantation (FMT). (**E**) 16S rRNA gene sequencing was employed to profile the gut microbiota and investigate compositional shifts in mice. (**F and G**) Immunofluorescence staining of small intestinal tuft cells. Tuft cells were labeled with Dclk1 (red) and counterstained with DAPI (blue) for nuclear visualization. The average number of cluster cells per individual villus was quantified (*n* = 6 per group). Scale bar: 40 μm. Experiments were repeated independently two times. Data are the mean ± SEM. Statistical significance was determined by two-sided Student’s *t* test (**B, F**).

DAT is known to be fermented by the gut microbiota, specifically *Flavonifractor plautii* (15). Studies indicate that DAT administration influences the alpha diversity index of the gut microbiota (16). To determine whether the gut microbiota is essential for DAT-induced tuft cell proliferation in the small intestine, mice underwent a 7-day course of broad-spectrum antibiotics to deplete gut microbiota (Fig. 4A). Strikingly, DAT failed to induce tuft cell proliferation in microbiota-depleted mice compared to untreated controls (Fig. 4B and C).

To further validate this dependency, we performed fecal microbiota transplantation (FMT). Antibiotic-treated mice received microbiota from either water-treated (control) or DAT-treated donors. To further validate this dependency, we performed fecal microbiota transplantation (FMT). Antibiotic-treated mice received microbiota from either water-treated (control) or DAT-treated donors (Fig. 4D). To investigate whether DAT administration alters the gut microbiota and whether such changes can be transmitted via fecal microbiota transplantation (FMT) to elicit consistent phenotypic effects, 16S rRNA sequencing was performed. The results revealed that DAT treatment significantly reshaped the gut microbial structure in mice, characterized by a prominent enrichment of the family *Erysipelotrichaceae* and a concurrent reduction in the abundance of *Lactobacillus* (Fig. 4E). Immunofluorescence analysis revealed that the number of intestinal cluster cells was increased in mice treated with DAT followed by fecal microbiota transplantation (Fig. 4F and G). Collectively, these findings demonstrate that the gut microbiota is indispensable for mediating DAT-induced tuft cell expansion in the small intestine.

### DAT promotes tuft cell through HDAC3

**Fig 5:**
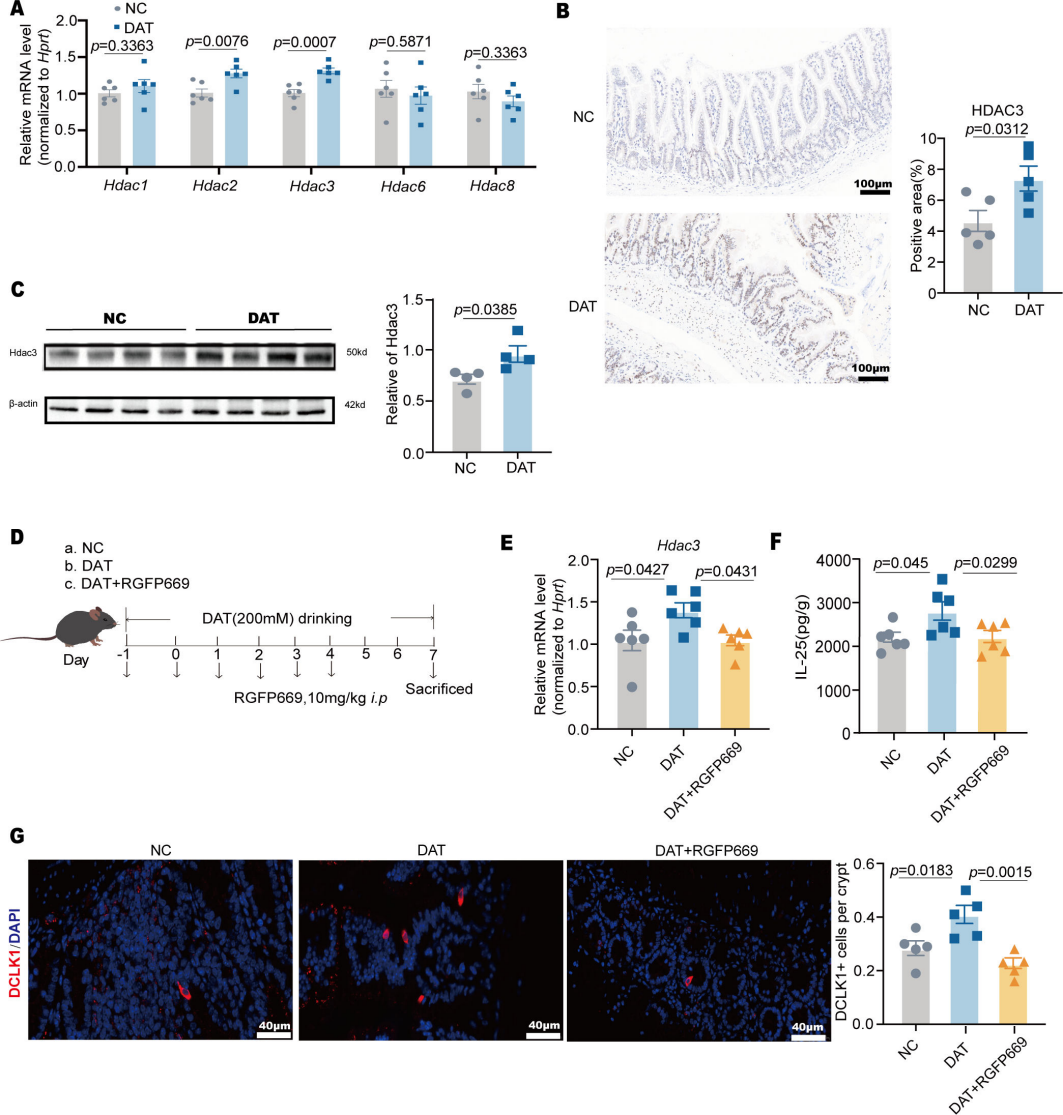
DAT promotes tuft cell through HDAC3. (**A**) Quantification of gene expression in the ileocecal region of the small intestine, including *Hdac1, Hdac2, Hdac3, Hdac6,* and *Hdac8* via RT-qPCR (*n* = 6 per group). (**B**) Immunohistochemical analysis of HDAC3 expression in small intestinal tissue (*n* = 5 per group). Scale bar: 100 μm. (**C**) Western Blotting analysis of small intestinal tissue: Hdac3 and β-actin staining (*n* = 4 per group). (**D**) Animal model establishment by administering the HDAC3 inhibitor RGFP669. According to the experimental design and treatment timeline, mice were treated with RGFP669 to inhibit HDAC3 activity in the small intestine. (**E**) RT-qPCR analysis of *Hdac3* gene expression in the small intestine following RGFP669 administration (*n* = 6 per group). (**F**) Measurement of IL-25 cytokine secretion from small intestinal tuft cells using enzyme-linked immunosorbent assay (ELISA) (*n* = 6 per group). (**G**) Immunofluorescence staining of small intestinal tuft cells. Cells were labeled with Dclk1 (red) and counterstained with DAPI (blue) for nuclear visualization. The average number of tuft cells per individual crypt was quantified (*n* = 5 per group). Scale bar: 40 μm. Experiments were repeated independently two times. Data are the mean ± SEM. Statistical significance was determined by two-sided Student’s’ *t* test (**A–C**) or one-way ANOVA (**E–G**).

Global analyses of histone acetylation in intestinal epithelial cells (IECs) revealed both increased and decreased histone acetylation at genes involved in diverse and essential pathways in the ileum of mice following butyrate administration (23). Similarly, HDAC3 expression was elevated in the small intestine in DAT-treated mice compared to normal control (NC) mice (Fig. 5A through C), suggesting that DAT enhances histone deacetylation in small intestine.

Previous studies have shown that HDAC3 is essential for tuft cell homeostasis (24). To investigate whether DAT promotes tuft cell proliferation via HDAC3 activation, we inhibited HDAC3 using a specific inhibitor (RGFP669) (Fig. 5D and E). RGFP966 is a highly selective HDAC3 inhibitor and shows no inhibition to other HDACs (25). Our data indicate that HDAC3 inhibition reduced tuft cell-derived cytokines, such as IL-25 (Fig. 5F), and suppressed tuft cell prefoliation during DAT administration (Fig. 5G). These findings collectively suggest that DAT enhances the tuft cell prefoliation through HDAC3 and that HDAC3 is crucial for tuft cell homeostasis (26).

### DAT directs effective worm clearance

**Fig 6:**
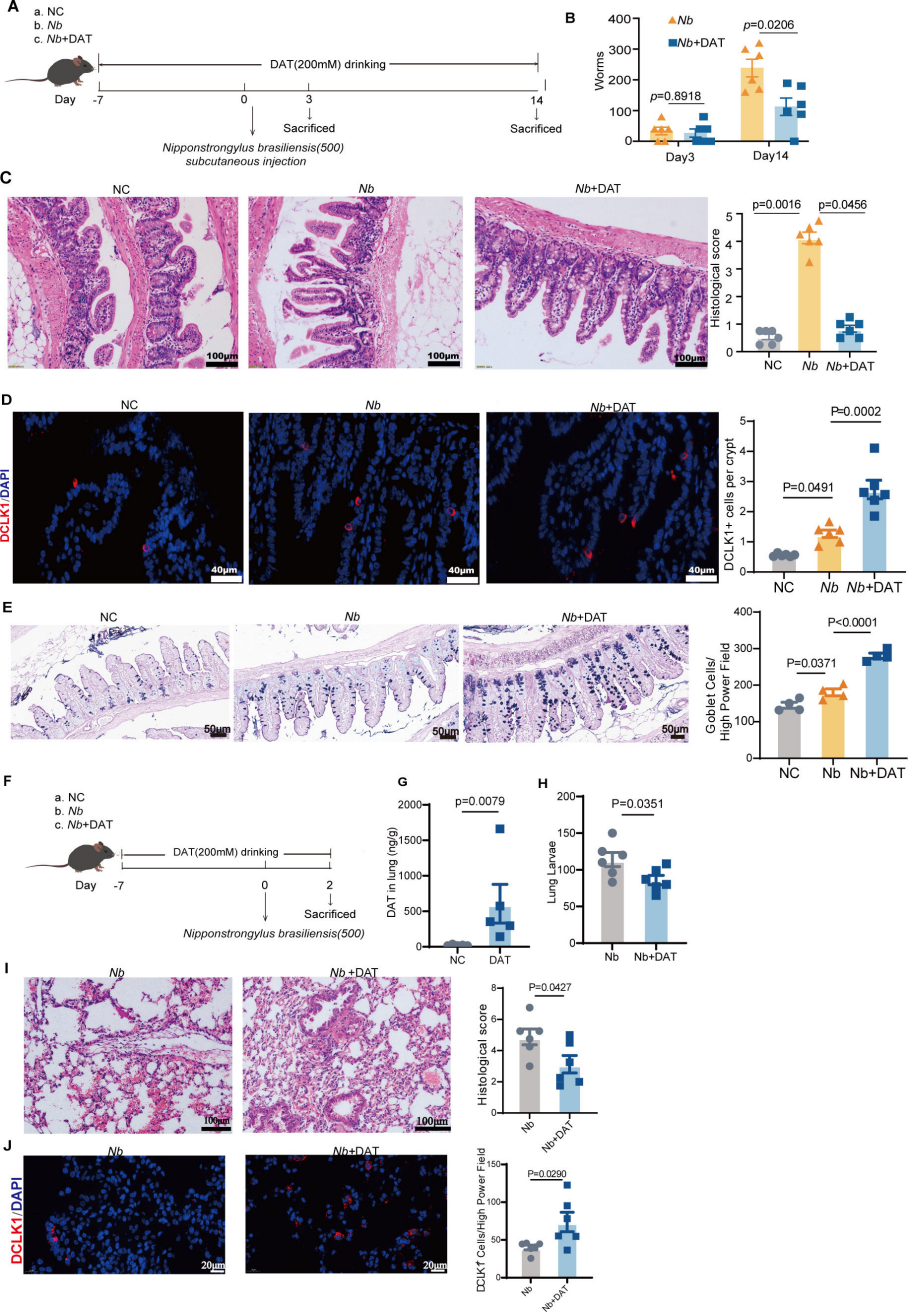
DAT directs effective worm clearance. (**A**) A schematic diagram of the experimental approach of mice infected with *Nipponstrongylus brasiliensis* (Nb) in DAT treatment model. (**B**). Worm counts were performed in the small intestine on days 3 and 14 post-infection (*n* = 6 per group). (**C**) HE staining was used to assess histopathological changes in small intestine tissue (*n* = 6 per group). Scale bar: 100 μm. (**D**) Immunofluorescence staining of small intestinal tuft cells. Tuft cells were labeled with Dclk1 (red) and counterstained with DAPI (blue) for nuclear visualization. The average number of tuft cells per individual crypt was quantified (*n* = 6 per group). Scale bar: 40 μm. (**E**) Combined Alcian Blue-periodic acid Schiff (AB-PAS) staining was performed to detect goblet cells, average number of goblet cells per high-power field (HPF) (*n* = 4 per group). Scale bar: 50 μm. (**F**) A schematic diagram of the experimental approach of mice infected with *Nipponstrongylus brasiliensis* (Nb) in DAT treatment model. (**G**) Targeted metabolomics analysis of DAT levels in mouse lung. (**H**) Worm counts were performed in the lung on days 2 post-infection (*n* = 6 per group). (**I**) HE staining was used to assess histopathological changes in lung (*n* = 6 per group). Scale bar: 100 μm. (**J**) Immunofluorescence staining of lung tuft cells. Tuft cells were labeled with Dclk1 (red) and counterstained with DAPI (blue) for nuclear visualization. The average number of tuft cells per individual crypt was quantified (*n* = 6 per group). Scale bar: 20 μm. Experiments were repeated independently two times. Data are the mean ± SEM. Statistical significance was determined by non-parametric Mann-Whitney test (**B, G–J**), non-parametric Kruskal-Wallis test (**C–E**).

Our results above demonstrate that type 2 immune responses are regulated by a signaling circuit involving tuft cells and ILC2s following DAT intervention. Type 2 immunity functions as a defense mechanism against helminth infections (17, 27, 28). To evaluate the impact of DAT in helminth infection, mice were infected with *Nippostrongylus brasiliensis* (Nb), a nematode parasite that migrates from the skin through the lungs to the small intestine (Fig. 6A). DAT-exposed mice exhibited enhanced type 2 inflammation, as indicated by an accelerated expulsion of parasites (Fig. 6B). As a result, tissue damage was alleviated in the DAT-treated group compared to the Nb infected group, demonstrating a protective effect of DAT against the parasite (Fig. 6C). In line with our above findings, DAT further enhanced the proliferation of both tuft cells and goblet cells in the infection group (Fig. 6D and E). These data suggest that DAT confers protective immunity against parasitic infections in the small intestine.

The larval form of the parasite migrates through host tissues to the lungs, where it is subsequently coughed up, swallowed, and ultimately reaches the intestines within 3–4 days, completing the infection route (29). To investigate whether DAT can be systemically absorbed and modulate immune responses in peripheral tissues—particularly during the early stage of Nb lung infection—we assessed its distribution and efficacy. Our results indicate that DAT reaches detectable concentrations in the lung tissue, demonstrating its ability to disseminate to organs beyond the intestinal tract (Fig. 6G). Furthermore, the DAT-treated group exhibited a significant reduction in larval burden compared to controls, indicating that DAT contributes to the clearance of parasites in the lungs (Fig. 6H). Moreover, the DAT-treated group ameliorated infection-induced tissue damage and concurrently increased the number of tuft cells in the lung tissue (Fig. 6I and J). Our results suggest that DAT may promote parasite clearance in both the intestine and lungs potentially through enhancing type 2 immunity.

### Tuft cell-dependent DAT signaling defense against intestinal parasitism

**Fig 7:**
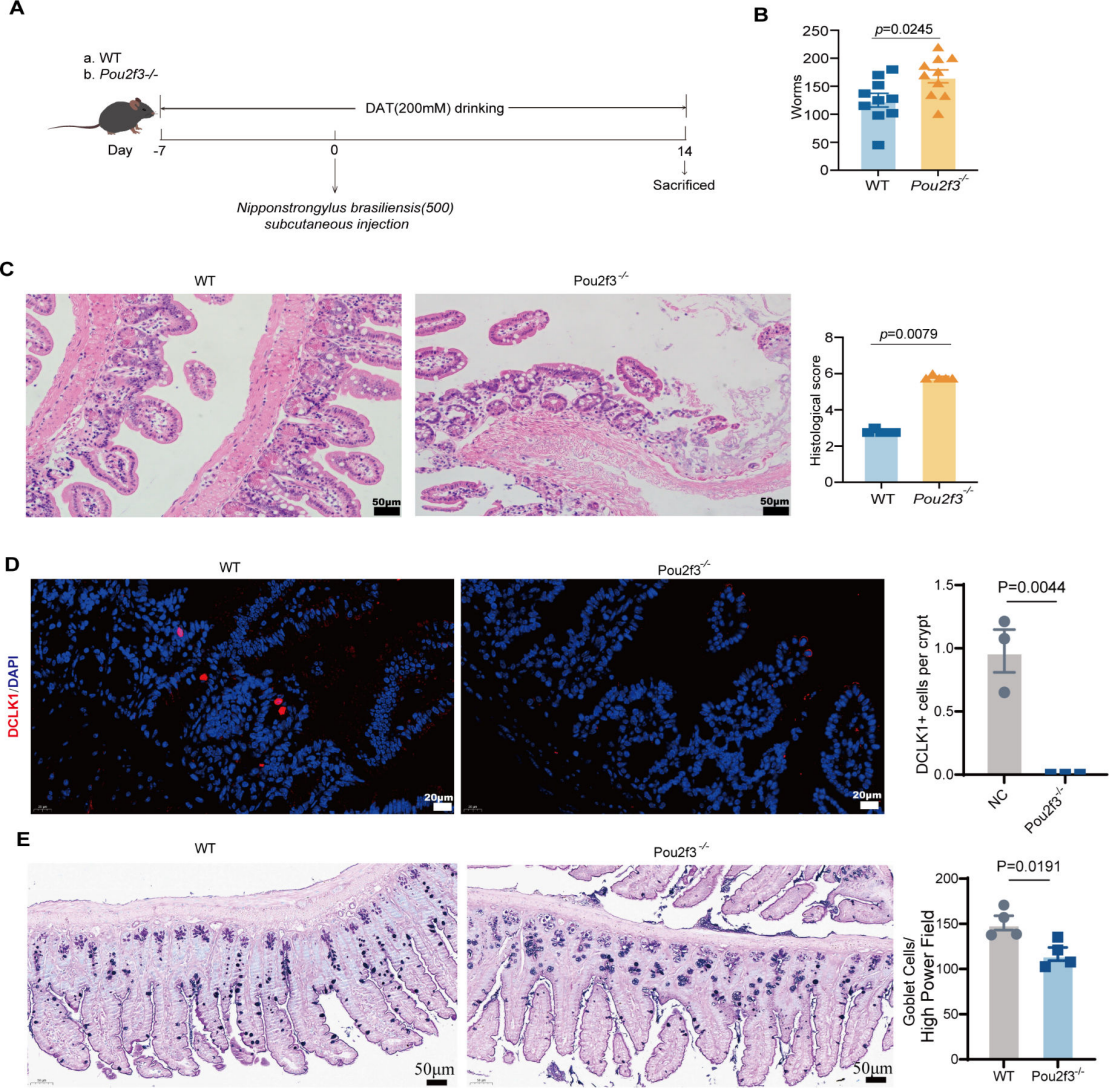
Tuft cell-dependent DAT signaling defense against intestinal parasitism. (**A**) A schematic diagram of the experimental approach of wild-type (WT) mice and tuft cell-deficient (Pou2f3^−/−^) mice infected with *Nipponstrongylus brasiliensis* (Nb) in DAT treatment model. (**B**) Worm counts were performed in the small intestine on day 14 post-infection (*n* = 10 per group). (**C**) HE staining was used to assess histopathological changes in small intestine tissue (*n* = 5 per group). Scale bar: 50 μm. (**D**) Immunofluorescence staining of small intestinal tuft cells. Tuft cells were labeled with Dclk1 (red) and counterstained with DAPI (blue) for nuclear visualization (*n* = 3 per group). Scale bar: 20 μm. (**E**). Combined Alcian Blue-periodic acid Schiff (AB-PAS) staining was performed to detect goblet cells, average number of goblet cells per high-power field (HPF) (*n* = 4 per group). Scale bar: 50 μm. Experiments were repeated independently two times. Data are the mean ± SEM. Statistical significance was determined by non-parametric Mann-Whitney test (**B–E**).

To investigate whether DAT mediates host resistance against intestinal parasitic infection through tuft cells, we established a parasite infection model using both wild-type (WT) mice and tuft cell-deficient (*Pou2f3^−/^*^−^) mice, comparing infection outcomes between groups (Fig. 7A).

At 14 days post-infection, intestinal parasite burden was significantly lower in WT mice than in *Pou2f3^−/^*^−^, indicating a critical role of tuft cells in DAT anti-parasitic defense (Fig. 7B). Histopathological analysis (H&E staining) revealed preserved mucosal integrity with minimal inflammation and epithelial damage in WT mice, whereas *Pou2f3^−/−^* mice exhibited severe mucosal disruption and inflammatory infiltration (Fig. 7C).

Immunofluorescence staining for DCLK1 (a tuft cell marker) confirmed significantly increased DCLK1^+^ tuft cells in infected WT intestines, with an inverse correlation between DCLK1^+^ cell density and parasite load. *Pou2f3^−/−^* mice showed undetectable DCLK1^+^cells (Fig. 7D), demonstrating that tuft cell deficiency impairs parasite clearance. Corroborating this finding, histological analysis by AB-PAS staining showed a marked reduction in goblet cell population in knockout mice compared to control animals after the same DAT treatment and parasite challenge, highlighting the essential function of goblet cells as a primary defense mechanism for intestinal protection (Fig. 7E).

In summary, DAT enhances anti-parasitic immunity by activating tuft cells in a Pou2f3-dependent manner, thereby reducing parasite colonization and maintaining barrier integrity. Tuft cells deficiency disrupts this protection, rendering *Pou2f3^−/−^* mice highly susceptible to infection.

## DISCUSSION

**Fig 8:**
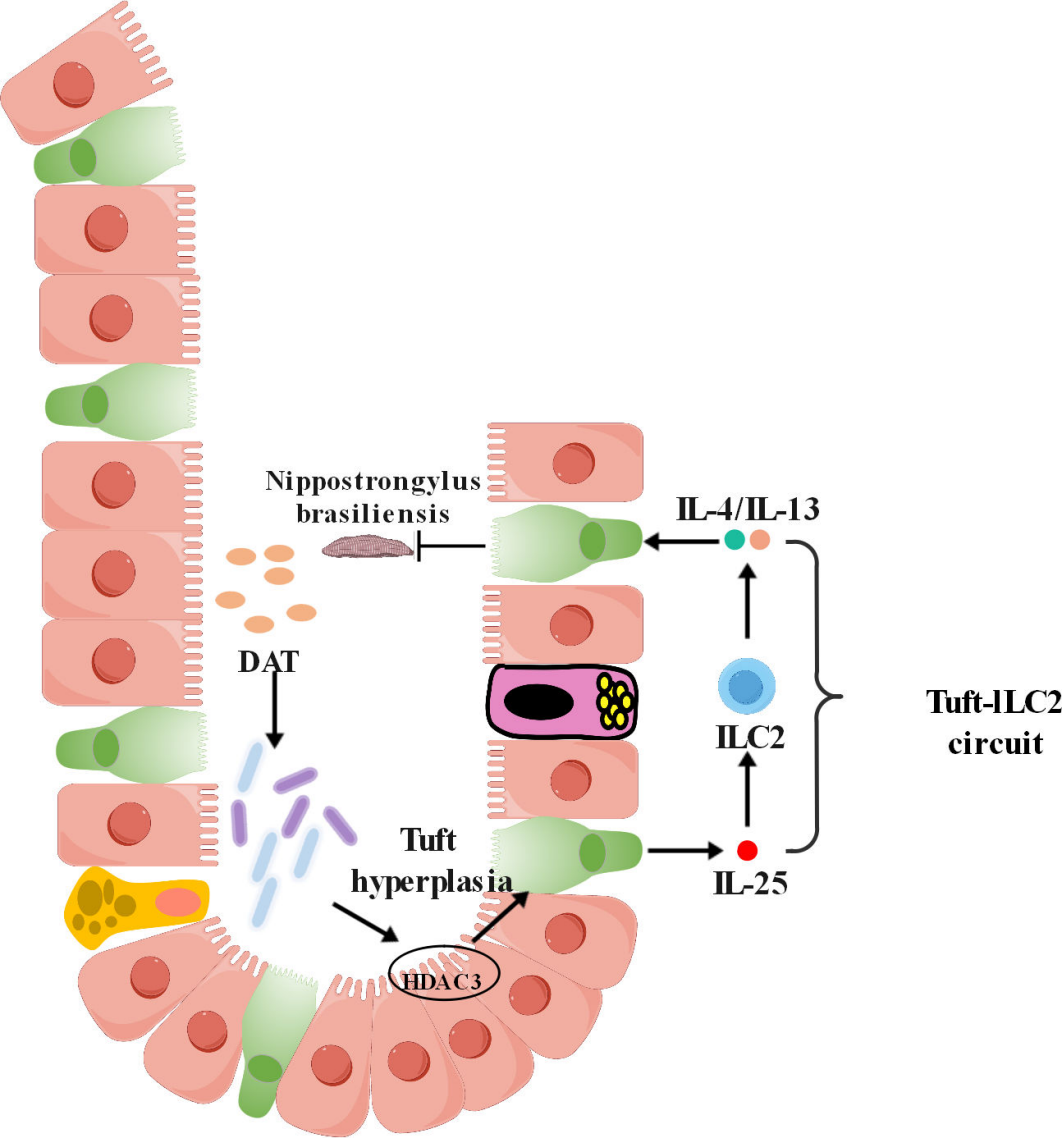
The hypothetical model of how DAT promotes tuft cell expansion and integrates into intestinal type 2 immunity.

This study demonstrates that desaminotyrosine (DAT), a microbiota-derived metabolite, potently stimulates tuft cell expansion in the small intestine. We further identify HDAC3 as a critical mediator through which DAT drives tuft cell accumulation. By modulating tuft cell numbers, DAT enhances the secretion of IL-25, which, in turn, activates group 2 innate lymphoid cells (ILC2s), leading to amplified type 2 immunity—a key defense mechanism against helminth infections (Fig. 8). The ability of DAT to promote tuft cells proliferation reveals a novel mechanism for boosting immune responses, particularly against parasitic infections (30).

The ability of DAT to stimulate tuft cell activity and boost type 2 immune responses opens up new avenues for managing infections that rely on a robust immune defense. Since succinate is known to promote tuft cell-driven type 2 immunity (13), we investigated if DAT’s effects were mediated through altering succinate levels. The lack of a significant increase in intestinal succinic acid after DAT intervention demonstrates that its immune regulatory function may be succinate-independent. The head-to-head experiment comparing DAT and succinic acid also revealed that the promoting effect of DAT on the expansion of intestinal tuft cells was weaker than that of succinic acid. However, the effect of DAT on the expansion of intestinal tuft cells persisted even after removing DAT for 3 days, indicating that DAT regulates the stability of tuft cell expansion. Here, we have discovered a new type of immunomodulatory effect of flavonoids or the metabolite DAT produced by gut bacteria, which can serve as an alternative to succinate metabolites.

Recent advances have underscored the role of intestinal tuft cells as central regulators of immune homeostasis. These cells act as sensors of microbial signals and play pivotal roles in orchestrating immune activation and tissue repair (20). Our work highlights DAT, a flavonoid-derived bacterial metabolite, as a potent modulator of this circuit. Notably, while exogenous DAT administration robustly induced tuft cell hyperplasia and type 2 immunity, this effect was abolished in microbiota-depleted mice. Furthermore, when the fecal microbiota from the DAT intervention was transplanted into mice after antibiotic clearance, it also showed the same promotion of tuft cell proliferation. This indicates that the DAT intervention may exert its effect through the intestinal microbiota. Further sequencing analysis of the microbiome revealed that DAT alters the composition of the intestinal microbiome, and the mice that received fecal transplants under DAT intervention also exhibited the same changes in the dominant bacterial populations, such as the enrichment of the family *Erysipelotrichaceae* and the reduction of *Lactobacillus* bacteria. Future study is of crucial importance for identifying the specific bacterial communities involved in the DAT process.

The immunomodulatory properties of DAT present a promising therapeutic strategy for parasitic diseases. DAT induces tuft cells expansion and consequently activates ILC2s. Activated ILC2s produce type 2 cytokines, such as IL-4 and IL-13, which orchestrate the immune response against parasitic invaders, contributing to epithelial regeneration and immune defense (9, 31). During parasitic infections like helminthiasis, the regenerative capacity of tuft cells facilitates rapid intestinal repair, restoring homeostasis and maintaining immune surveillance. Therefore, by enhancing tuft cell function, DAT may play a vital role in strengthening this natural defense mechanism. Given that *Nippostrongylus brasiliensis* migration involves the lungs before reaching the gut (29), it is crucial to know if oral DAT has systemic immunological impacts beyond the gut. To address this important question, we performed experiments assessing immune responses in lung tissues during the early phase of parasite migration. In particular, our results revealed that DAT was capable of eliminating larvae in the lungs, protecting lung tissue from pathological damage, and promoting the proliferation of tuft cells in the lungs. In summary, we have confirmed that the systemic availability of DAT not only affects intestinal immune regulation but also has a role in the expansion of tuft cells in the lungs and the clearance of larvae, which clarify these potential systemic effects.

HDAC3 serves as a central link between the intestinal microbiota and diverse intestinal physiological processes. Epithelial HDAC3 regulates intestinal epithelial and immune homeostasis, as well as, susceptibility to bacterial infection and obesity (32, 33). Following reports that microbiota-derived butyrate restricts tuft cells differentiation via HDAC3 (34), our findings demonstrate that DAT activates the tuft cell-ILC2 pathway through HDAC3 to modulate intestinal type 2 immunity. This divergence highlights the context-dependent roles of HDAC3 and microbial metabolites in shaping epithelial and immune responses. Further investigation is needed to elucidate how DAT engages HDAC3.

Despite its promising effects in modulating tuft cell function and enhancing immune responses, the clinical applications of DAT remain largely unexplored. Further studies are required to understand the molecular pathways through which DAT activates tuft cells and how this interaction can be harnessed for therapeutic purposes. The potential of DAT as a treatment for parasitic infections, particularly in clinical settings, warrants comprehensive investigation, including preclinical and clinical trials to evaluate its safety, efficacy, and long-term effects.

DAT is associated with the development of several diseases including influenza, cancer (35), and IBD. Nonetheless, the findings described here reveal a central role for DAT in epigenetically promoting type 2 intestinal immune responses through regulation of tuft cells expansion. By targeting tuft cells, DAT may provide a novel therapeutic approach for infections requiring a robust type 2 immune response, such as helminth infections. With further exploration, DAT could emerge as a valuable tool for managing immune-mediated disorders and parasitic infections, ultimately improving clinical outcomes (36).

## MATERIALS AND METHODS

### Animals

Wild-type male C57BL/6J mice, aged 6–8 weeks, were purchased from Xuzhou Medical University. The C57BL/6J *Pou2f3^−/−^* male mice, also aged 6–8 weeks, were provided by Professor Yugang Wang at Xuzhou Medical University. All mice were bred and maintained under specific pathogen-free (SPF) conditions. They were housed in a 12-h alternating light-dark cycle, with access to standard laboratory sterilized feed and water *ad libitum*. The room temperature was maintained at 25°C, with humidity controlled between 40% and 70%.

### DAT treatment experiment

Mice were randomly divided into two groups: (i) normal control (NC) mice were provided sterile water as drinking water for 7 days; (ii) DAT group mice were given the 200 mM DAT (BD6690, Bidepharm) solution as drinking water for 7 days. On day 7, all mice were sacrificed to assess the effects of DAT treatment. (iii) DAT (remove) group: mice were given the 200 mM DAT (BD6690, Bidepharm) solution as drinking water for 7 days; after 7 days, DAT was discontinued and replaced with normal drinking water for 3 days.

### Succinic acid treatment experiment

Mice were randomly divided into two groups: (i) normal control (NC) mice were provided sterile water as drinking water for 7 days; (ii) succinic acid group mice were given the 150 mM succinic acid (A100165-0500, Diamond) solution as drinking water for 7 days. On day 7, all mice were sacrificed to assess the effects of succinic acid treatment.

### Hdac3 inhibition experiment

Mice were assigned to three groups: (i) normal group mice received sterile water as drinking water for 7 days; (ii) DAT group mice were administered the 200 mM DAT solution as drinking water for 7 days; (iii) DAT + RGFP966 group mice were intraperitoneally injected with the Hdac3 inhibitor RGFP966 (HY-13909, Med Chem Express) 15 mg/kg one day before the start of DAT treatment. Hdac3 inhibitor RGFP966 was administered daily for 5 consecutive days (37). Concurrently, mice were provided the 200 mM DAT solution as drinking water for 7 days. The effects of Hdac3 inhibition were evaluated following this treatment protocol.

### Nippostrongylus brasiliensis infection experiment

Mice were divided into three groups: (i) mice received sterile water as drinking water throughout the study (NC group), (ii) mice received a subcutaneous injection of 500 *N*. *brasiliensis* larvae on day 0 (Nb group), (iii) mice received a subcutaneous injection of 500 *N*. *brasiliensis* larvae on day 0 and were provided a 200 mM DAT solution as drinking water starting from day −7 for 14 days (Nb + DAT group). Mice were sacrificed on day 3 and day 14 to evaluate the effects of DAT intervention on *N. brasiliensis* infection.

Mice were divided into two experimental groups: (i) wild-type (WT) mice subcutaneously injected with 500 *N. brasiliensis* (Nb) at day 0 and administered 200 mM DAT in drinking water from day −7 to day 14 (WT + Nb + DAT group); (ii) *Pou2f3^−/−^* mice subcutaneously injected with 500 Nb larvae at day 0 and administered 200 mM DAT in drinking water from day −7 to day 14 (*Pou2f3^−/−^* + Nb + DAT group). All mice were euthanized at day 14 post-infection to evaluate the role of tuft cells in DAT-mediated protection against *N. brasiliensis* infection.

### Intestinal microbiota depletion experiment

Mice were randomly assigned to four groups: NC, DAT, ABX, and ABX+DAT. From days 0 to 7, the NC and DAT groups were provided normal water with no interventions, while the ABX and ABX + DAT groups received broad-spectrum antibiotics ampicillin 1 mg/ml (A100339, Diamond, China), neopenicillin sulfate 1 mg/ml (1406-41-0, Sangon Biotech, China), metronidazole 1 mg/ml (A600633, Sangon Biotech, China), vancomycin 0.5 mg/ml (A600983, Sangon Biotech, China) for 7 days. Antibiotics were replaced every 3 days to maintain their activity. From days 7 to 14, the NC group continued to receive normal water, the DAT group was given DAT 200 mM, while the ABX + DAT group was administered both DAT 200 mM and broad-spectrum antibiotics simultaneously.

### Fecal microbiota transplantation experiment

Mice were randomly assigned to two groups: donor mice and recipient mice. The donor mice were further divided into the NC and DAT groups. The NC group received normal water for 7 days, while the DAT group was provided with DAT 200 mM water for the same duration. After 1 week of intervention, feces were collected from both the NC and DAT groups. The fecal samples were then used for fecal microbiota transplantation (FMT) in recipient mice, labeled FMT-NC and FMT-DAT, respectively.

Donor feces were collected and weighed, and 200 mg of feces was dissolved in 1 mL of 0.05% cysteine (30089, Sigma, USA) solution. The mixture was thoroughly stirred and filtered through a 100 μm screen, and the resulting fecal filtrate was used for transplantation. Each recipient group was gavaged with 200 μL of the corresponding fecal filtrate for 6 consecutive days, with samples collected on the 7th day.

### RNA extraction and RT-qPCR

RNA was extracted from small intestine tissues using Total RNA was extracted using the Total RNA Extraction Kit (R1200, Solarbio, Beijing, China) following the manufacturer’s protocol. Complementary DNA (cDNA) was synthesized using the Prime Script RT Reagent Kit (G3330, Servicebio, China). Reverse transcription-quantitative polymerase chain reaction (qRT-PCR) was subsequently performed using SYBR Green qPCR Master Mix (b21203, Bimake, USA) on a 7900 Fast Real-Time PCR system (Roche Switzerland). The thermal cycling conditions were as follows: an initial denaturation at 95°C for 10 min, followed by 40 cycles of 95°C for 10 s, 60°C for 30 s, and 72°C for 32 s. Relative gene expression was calculated using the 2^−ΔΔCT^ method, with *Hprt* as the endogenous control. All target gene transcription levels were normalized to *Hprt*. Primer sequences are provided in Data S1.

### Identification and quantification of goblet cells in small intestinal

Goblet cells in small intestinal tissues were identified using periodic acid–Schiff (PAS) staining. Intestinal sections were fixed in 4% paraformaldehyde, dehydrated, and embedded in paraffin (10023418, SINOPHARM, China). The paraffin-embedded tissues were sectioned at a thickness of 5 µm, deparaffinized in xylene, and rehydrated through a graded ethanol series. The sections were oxidized in 0.5% periodic acid solution for 10 min, rinsed with distilled water, and stained with PAS dye solution B (G1049, Servicebio, China) for 15 min in the dark. After thorough washing, the sections were counterstained with hematoxylin for 1–2 min to visualize nuclei. The tissues were then dehydrated, cleared, and mounted with coverslips. Goblet cells were identified as magenta-stained cells under an Olympus DP74 microscope. Quantitative analysis was performed by counting PAS-positive goblet cells in high-magnification fields of view.

### Immunofluorescence

Paraffin-embedded tissue sections (4 μm thick) were deparaffinized by soaking in xylene three times, followed by rehydration through a graded ethanol series (100%, 100%, 95%, 90%, 80%, 70%) and rinsing in water. Antigen retrieval was performed by placing the sections in 0.1 M citrate buffer (pH 6.0) and heating them for 5 min. After cooling to room temperature, the sections were blocked with 5% bovine serum albumin (BSA) for 1 h. Primary antibody incubation was performed overnight at 4°C with anti-DCLK1 antibody (21699-1-AP, pProteintech, USA 1:800 dilution). Sections were washed five times with buffer and incubated with the appropriate secondary antibody (PV-6001, ZSGB-BIO, China) for 20 min at room temperature. Finally, the sections were counterstained with DAPI (C1005, Beyotime, China) to visualize nuclei. Images were acquired using a Tissue FAXS scanner (Tissue Gnostics, Austria). Fluorescence intensity was quantified using Image J software to ensure comparability across groups.

### Enzyme-linked immunosorbent assay

The small intestine of mice was removed, cleaned, and homogenized in RIPA lysis buffer (WB3100, New Cell & Molecular Biotech, China) containing a protease inhibitor cocktail (CW2200, CWBIO, China, 1:100 dilution). The homogenates were incubated on ice for 30 min and centrifuged at 10,000 × *g* for 10 min. The supernatants were collected and analyzed for IL-25, IL-4, IL-13, and IL-33 levels using the mouse uncoated ELISA Kit (Invitrogen, USA) according to the manufacturer’s instructions.

### Separation of lymphocytes

Mice were sacrificed, and approximately 8 cm of the ileocecal portion of the small intestine was removed. The intestinal sections were cut longitudinally, washed, and divided into one-centimeter segments before being placed in precooled PBS for further cleaning. Impurities were filtered through a 200-mesh nylon mesh, leaving the cleaned intestinal segments. These segments were then digested in 5 mL of a digestion solution:prepared by combining 1.2 mL of 500 mM EDTA, 1.2 mL of 1 M HEPES (C0217, Beyotime, China), and PBS to a total volume of 60 mL. Epithelial cells were dissociated by incubating the segments on a shaker at 37°C and 200 rpm. Next, the lamina propria tissues were finely minced and digested in RPMI 1640 medium containing 5% fetal bovine serum (HY-T1000, ExCell Bio, China), 1 mg/mL collagenase (11088866001, sigma, USA), 1 mg/mL hyaluronic acid (935166, sigma, USA), and 1 μg/mL DNase I (D806930, MACKLIN, China). The digestion was performed at 37°C and 100 rpm for 30 min. After incubation, the digested solution was filtered through a 70-μm cell strainer to obtain single-cell suspensions. The cells were resuspended in 40% percoll and subjected to centrifugation at 1,500 rpm for 15 min at 4°C to perform percoll gradient separation, isolating lamina propria lymphocytes. The resulting cell pellets were washed with cold PBS and resuspended in PBS containing 2% fetal bovine serum (FBS) (38).

### Flow cytometry

To analyze innate lymphoid cells 2 (ILC2s) and innate lymphoid cells 3 (ILC3s), 2 × 10^6^ cells were stained with a panel of antibodies targeting lineage markers conjugated to FITC (anti-CD45R, CD11c, Gr-1, TCR β chain, TCR γ/δ, FcεR1α, CD4, F4/80; 103205, 117305, 108405, 109205, 118105, 134305, 100510, 123108; Biolegend, USA 1:100 dilution). Additional staining included anti-CD45 conjugated to APC/Fire 750 (103153, 30-F11, Biolegend, USA, 1:100 dilution) and anti-CD127 (IL-7Rα) conjugated to PE/Cyanine7 (135013, A7R34, Biolegend, USA, 1:100 dilution). The staining was performed for 50 min at 4°C. Following surface marker staining, cells were fixed with True Nuclear Fix solution (424401, Biolegend, USA) for 1 h and subsequently permeabilized with True Nuclear Perm buffer (424401, Biolegend, USA). Intracellular staining was performed using anti-GATA3 conjugated to PE (653803, 16E10A23, Biolegend, USA, 1:20 dilution) and anti-RORγ(t) conjugated to PerCP-eFluor 710 (46-6981-80, B2D, eBioscience, USA, 1:100 dilution) for 1 h. Flow cytometry was conducted on a FACS Canto II Flow Cytometer (BD Biosciences, USA), and data were analyzed using FlowJo software.

### Western blotting

Ileum tissue from the small intestine was excised, homogenized by grinding with RIPA lysis buffer and sonicated. The sample was then centrifuged at 1,000 × *g* for 10 min at 4°C. The supernatant was collected for protein quantification using BCA protein assay kit (KGB2101-500, KeyGEN BioTECH, Nanjing, China). Equal amounts of protein were subsequently loaded onto an SDS-PAGE gel (P2012, New Cell & Molecular Biotech, Suzhou, China) for electrophoresis, followed by transfer to PVDF membranes (IPVH0010, MERCK, USA). Membranes were blocked with 5% skim milk powder (VIC1411, VICMED, Xuzhou, China) in PBS for 1 h at room temperature to prevent non-specific binding. The membranes were then incubated overnight at 4°C with primary antibodies: β-actin (66009, Proteintech, USA, 1:10,000 dilution), Dclk1 (21699-1-AP, Proteintech, USA, 1:800 dilution), and anti-HDAC3 (AB76295, Abcam, England, 1:2,000 dilution). On the following day, the membrane was washed and incubated together with the corresponding secondary antibody using the universal antibody diluent (VP6022-500, VICMED, Xuzhou, China). Finally, ECL reagent (P10300, New Cell & Molecular Biotech, Suzhou, China) was added for chemiluminescent detection and analyzed using Fiji software(28).

### 16S rRNA gene sequencing

16S rRNA gene sequencing was performed on fresh murine small intestinal content samples. Total DNA was extracted using the FastPure Stool DNA Isolation Kit (MJYH, Shanghai, China) according to the manufacturer’s instructions. The V3–V4 regions of the 16S rRNA gene were amplified with primers 338F and 806R. Purification was carried out with a DNA Gel Extraction Kit (YuHua, China). Libraries were prepared with the NEXTFLEX Rapid DNA-Seq Kit. Raw reads were quality-controlled and assembled using fastp (v0.19.6) and FLASH (v1.2.11), respectively. OTUs were clustered at 97% similarity with UPARSE (v7.1). Samples were rarefied to 20,000 sequences per sample (average coverage: 99.09%). Taxonomic classification was conducted using the RDP Classifier (v2.11) against the SILVA database (v138) at a 70% confidence threshold. Microbial community composition and functional potential (predicted via PICRUSt2 v2.2.0) were analyzed. All bioinformatic processing was performed on the Majorbio Cloud Platform.

### Targeted metabolomics analysis

DAT and succinic acid analysis was performed using ultra-performance liquid chromatography-tandem mass spectrometry (UPLC-MS/MS). Metabolites from serum, cecal content, and lung tissue were extracted with methanol/water (80%), followed by vortexing, centrifugation, lyophilization, and reconstitution in 50% methanol. Chromatographic separation employed a gradient of 0.1% formic acid in water (A) and 0.1% formic acid in acetonitrile (B) at 0.3 mL/min. MS detection used negative ESI mode with MRM. Calibration standards (10–10,000 ng/mL) were prepared from 100 μg/mL stock. Linear regression (unweighted, *R*² > 0.99) was used for quantification. LOD and LOQ were defined as S/N ratios of 3 and 10, respectively.

### Gross and histological assessment

The histopathological evaluation of mouse lung tissues stained with hematoxylin and eosin (H&E) was performed using a semi-quantitative scoring system based on two parameters: inflammation and tissue damage. The inflammation score was graded as follows: 0 (absent), 1 (mild, with focal infiltration of lymphocytes and eosinophils), 2 (moderate, with diffuse inflammatory cell infiltration and occasional eosinophilic microabscesses), and 3 (severe, with extensive eosinophilic abscesses, granuloma formation, and/or tissue necrosis). Tissue damage was scored as: 0 (normal architecture), 1 (mild alveolar septal thickening or minor hemorrhage), 2 (moderate alveolar destruction or early interstitial fibrosis), and 3 (severe tissue necrosis, structural collapse, or marked fibrosis).

The histopathological evaluation of mouse small intestine sections stained with hematoxylin, and eosin was performed using a semi-quantitative scoring system based on three parameters: epithelial tissue damage, mucosal swelling, and inflammatory cell infiltration. Each parameter was graded on a scale of 0 to 3. Epithelial damage was scored as: 0 (intact structure), 1 (focal loss of superficial epithelial cells), 2 (discontinuity of the epithelium involving <50%), and 3 (extensive epithelial loss involving ≥50%). Mucosal swelling was scored as: 0 (absent), 1 (mild), 2 (moderate), and 3 (severe). Inflammatory cell infiltration was graded as: 0 (no neutrophils), 1 (a few inflammatory cells confined to the lamina propria), 2 (diffuse inflammatory cells distributed throughout the mucosa), and 3 (inflammatory cells transmurally extending into the muscular layer).

### Statistical analysis

Statistical analyses were performed using GraphPad Prism version 6.0 (GraphPad software, San Diego, CA). Data were presented as mean ± standard error of the mean (SEM). Significance in all cases was set at *P <* 0.05. Scores, weights, and assay values were analyzed using a one-way ANOVA test, non-parametric test where appropriate. With bacterial quantification assays, the log_10_ values of the results were used to achieve normal distribution.

## Data Availability

All data generated in this study and included in this article are available from the corresponding author on reasonable request.The 16S rRNA-seq data reported in this paper have been deposited in the Genome Sequence Archive in National Genomics Data Center, China National Center for Bioinformation/Beijing Institute of Genomics, Chinese Academy of Sciences (GSA: CRA036511) that are publicly accessible at https://ngdc.cncb.ac.cn/gsa/browse/CRA036511.

## SUPPLEMENTAL MATERIAL

Fig. S1 mbio.03289-25-s0001.pdf The effect of DAT on promoting tuft cell proliferation compared with succinic acid.

Supplemental material mbio.03289-25-s0002.docx Fig. S1 legend and Data S1.

ASM does not own the copyrights to Supplemental Material that may be linked to, or accessed through, an article. The authors have granted ASM a non-exclusive, world-wide license to publish the Supplemental Material files. Please contact the corresponding author directly for reuse.
