# Supplementary figures and images for: Desaminotyrosine promotes tuft cell expansion and integrates intestinal type 2 immunity
Source: mBio. 2026 Jan 23;17(2):e03289-25. doi: 10.1128/mbio.03289-25 (PMC12893008; doi:10.1128/mbio.03289-25)

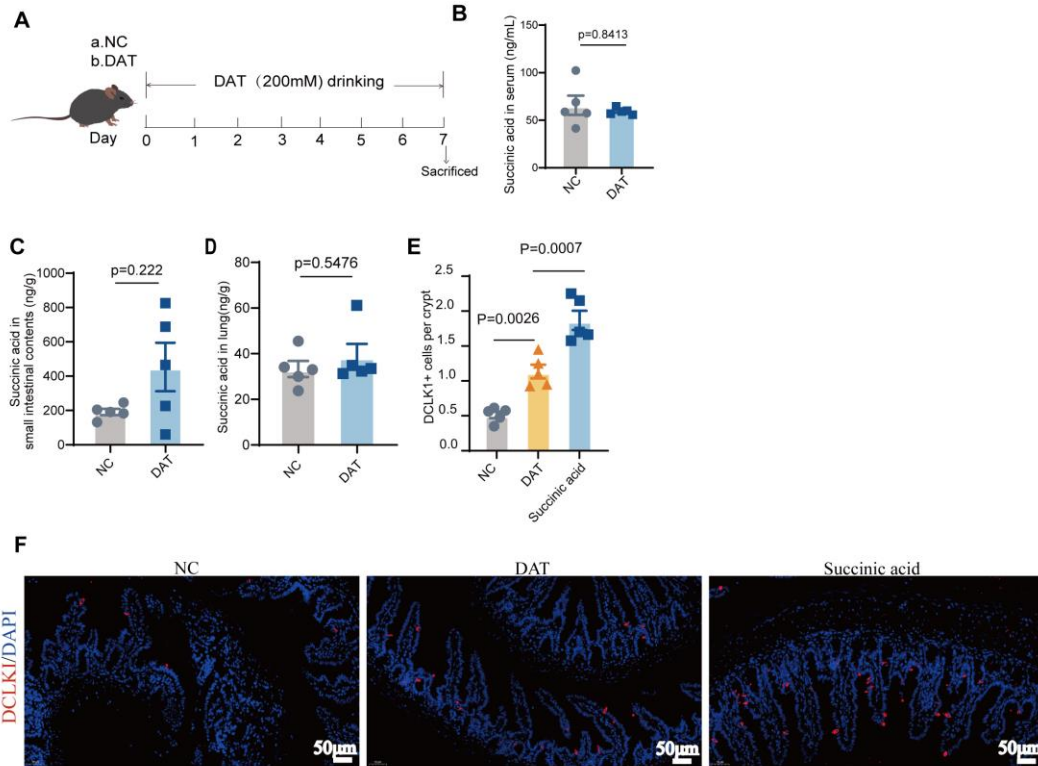

Supplement: Fig. S1 — The effect of DAT on promoting tuft cell proliferation compared with succinic acid. [file mbio.03289-25-s0001.pdf]
