# Supplementary material for: Desaminotyrosine promotes tuft cell expansion and integrates intestinal type 2 immunity
Source: mBio. 2026 Jan 23;17(2):e03289-25. doi: 10.1128/mbio.03289-25 (PMC12893008; doi:10.1128/mbio.03289-25)
Supplement: Supplemental material — Fig. S1 legend and Data S1. [file mbio.03289-25-s0002.docx]

**Supplementary Fig.1. The effect of DAT on promoting tuft cell proliferation compared with succinic acid.** (A). Schematic of the experimental design. (B-D). Targeted Metabolomics Analysis of succinate levels in mouse serum, small intestinal contents, and lung tissue. (E-F). Fluorescence staining of tuft cells (DCLK1+, red) in ileum and DCLK1+ tuft cells per crypt (n=5 per group). Scale bar: 50 μm.Experiments were repeated independently two times. Data are the mean ± SEM. Statistical significance was determined by two-sided Students’ t test (B-F).

Supplementary Data 1: Reference gene and target gene sequences

| 基因名称 | 引物序列5’ - 3’ |
| --- | --- |
| *Hprt* | F：TCAGTCAACGGGGGACATAAA |
|  | R：GGGGCTGTACTGCTTAACCAG |
| *Lgr5* | F：CCTACTCGAAGACTTACCCAGT |
|  | R：GCATTGGGGTGAATGATAGCA |
| *Ascl2* | F：AAGCACACCTTGACTGGTACG |
|  | R：AAGTGGACGTTTGCACCTTCA |
| *Muc2* | F：AGGGCTCGGAACTCCAGAAA |
|  | R：CCAGGGAATCGGTAGACATCG |
| *Tff3* | F：TTGCTGGGTCCTCTGGGATAG |
|  | R：TACACTGCTCCGATGTGACAG |
| *Dclk1* | F：TCCACCGGAATTGAACTCGG |
|  | R：GGGAGCGAACAGTCTCAGA |
| *Pou2f3* | F：CTGGAACAGTAACGTCATCCTG |
|  | R：AGTTCATTGCTGCTTTGGAGTT |
| *IL-25* | F：ACAGGGACTTGAATCGGGTC |
|  | R：TGGTAAAGTGGGACGGAGTTG |
| *Trpm5* | F：CCAGCATAAGCGACAACATCT |
|  | R：GAGCATACAGTAGTTGGCCTG |
| *Chga* | F：ATCCTCTCTATCCTGCGACAC |
|  | R：GGGCTCTGGTTCTCAAACACT |
| *Lyz1* | F：GAGACCGAAGCACCGACTATG |
|  | R：CGGTTTTGACATTGTGTTCGC |
| *Mptx2* | F：AGACCTGACTCGCCCTTATAG |
|  | R：GGTGCTTTGAAACTGACTCCC |
| *Hdac1* | F：AGTCTGTTACTACTACGACGGG |
|  | R：TGAGCAGCAAATTGTGAGTCAT |
| *Hdac2* | F：GGAGGAGGCTACACAATCCG |
|  | R：TCTGGAGTGTTCTGGTTTGTCA |
| *Hdac3* | F：CACCAAGAGCCTTGATGCCTT |
|  | R：GCAGCTCCAGGATACCAATTACT |
| *Hdac6* | F：TCCACCGGCCAAGATTCTTC |
|  | R：CAGCACACTTCTTTCCACCAC |
| *Hdac8* | F：ACTATTGCCGGAGATCCAATGT |
|  | R：CCTCCTAAAATCAGAGTTGCCAG |
